# A High-Sensitivity Hydraulic Load Cell for Small Kitchen Appliances

**DOI:** 10.3390/s100908452

**Published:** 2010-09-09

**Authors:** Roman Pačnik, Franc Novak

**Affiliations:** 1 BSH Hišni aparati d.o.o., Savinjska cesta 30, 3331 Nazarje, Slovenia; 2 Jožef Stefan Institute, Jamova cesta 39, 1000 Ljubljana, Slovenia; E-Mail: franc.novak@ijs.si

**Keywords:** Hydraulic load cell, FEM, sensor, weighing

## Abstract

In this paper we present a hydraulic load cell made from hydroformed metallic bellows. The load cell was designed for a small kitchen appliance with the weighing function integrated into the composite control and protection of the appliance. It is a simple, low-cost solution with small dimensions and represents an alternative to the existing hydraulic load cells in industrial use. A good non-linearity and a small hysteresis were achieved. The influence of temperature leads to an error of 7.5%, which can be compensated for by software to meet the requirements of the target application.

## 1. Introduction

Load cells or force sensors are the heart of a weighing instrument. They are, in fact, force transducers that convert a load or a force into an electrical signal. The words “load” and “force” can be regarded as synonyms and are both used in industry and academia. There are various methods for measuring force [1]. However, among modern force sensors (load cells), by far the most commonly used method is to measure the strain produced in an elastic member by the unknown force. Typical representatives include strain-gauge-based load cells of various designs, *i.e.*, bending beam, shear beam, S-beam, canister, ring, button and others. In general, they cover a typical sensing range from 0.1 N to over 10^6^ N and their inaccuracy over the full scale (FS) is 0.003% to 1% [2,3]. Occasionally, the method of balancing the force against an electromagnetically developed force is used. These types of load cells are used when the highest accuracy is demanded, e.g., scales in special accuracy classes for precise weighing and laboratory use. Also, the method of converting the force to a liquid pressure and measuring that pressure is used in specific applications. Hydraulic load cells are force-balance devices, measuring weight as a change in the pressure of the internal filling fluid. In a rolling-diaphragm-type hydraulic load cell, a load or force acting on a loading head is transferred to a piston that, in turn, compresses a filling fluid confined within an elastomeric diaphragm chamber. Generally, they cover the sensing range from 50 N up to 5 × 10^7^ N and their inaccuracy over the full scale (FS) can be as low as 0.25% [2–4]. They are mainly used in industrial environments since they are compact, robust and reliable, even in the most hostile environments. With proper design of the hydraulic load cell the construction can be simplified, the inaccuracy improved and the creeping effect reduced [5].

The results presented in this paper stem from the design of a small kitchen appliance with a weighing function integrated into a control loop in order to prevent unbalanced movement of the appliance during operation. In this particular case, the weighing should be appropriate for larger amounts (up to 5 kg) of ingredients, e.g., flour. The size and shape of the load cells should be small, similar to the existing rubber feet of the appliance. In our case, four load cells will be used and integrated into the bottom of the appliance. The integrated load cells are loaded with the mass of the appliance (the dead load) as well as with the mass being weighed. On occasions, this dead load may be even greater than the actual load to be measured. Consequently, load cells for a higher measuring range must be used. Another requirement related to the target application is that small, low-cost load cells with high electrical output signals are employed. The goal was to develop a hydraulic load cell with a simple construction to cut down the costs. In addition, the load cell should be composed of standard components that can be easily assembled using environment-friendly technologies.

In fact it was the lack of devices complying with these requirements that fostered the development of the load cell reported in this paper. In particular, the following key parameters were considered in the analysis of the existing products: dimensions, sensitivity, robustness *vs.* device size and cost. A set of possible candidates were included in the comparison study. The results are summarised in a table in Section 4.

In this paper the developed hydraulic load cell made from hydroformed metallic bellows is presented. It has been implemented in a series of prototypes, the characteristics of which fulfill the requirements in such a manner that with some minor additional software corrections the desired functionality can be achieved.

## 2. Finite Element Method (FEM) Modelling and Simulation

### 2.1. Preliminary considerations

As mentioned above, we deal with four load cells integrated into the bottom of an appliance that weighs about 5 kg (dead load). During normal use the appliance is likely to be subjected to different shock loads imposed by the user. Consequently, the required load-cell capacity should be designed for the expected dynamic shock impact and can be calculated [3] with the following expression:

$$C=1.25K\frac{W_{T}+W_{N}}{N}=1.25\cdot 1.25\frac{5\text{kg}+5\text{kg}}{4}=4.68\text{kg}$$

where:

*C* = required load-cell capacity (kg)
*W*_T_ = tare weight (dead load) (kg)
*W*_N_ = net weight of projected vessel content (live load) (kg)
*N* = number of load cells
*K* = dynamic factor (in our case *K*=1.25)

**Figure 1.**
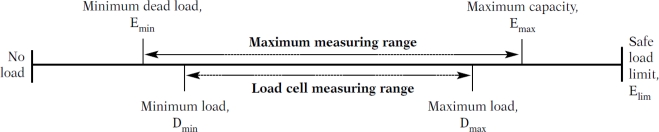
Graphical representation of the defined items.

For the purpose of the simulations the required load-cell capacity was taken to be 5 kg, which corresponds to the maximum capacity E_max_, as defined by recommendation of International Organization of Legal Metrology (Organisation Internationale de Métrologie Légale, OIML) [6]. The 5 kg of dead load divided by 4 (since we are dealing with 4 load cells) determines the minimum dead load E_min_ (*i.e.*, 1.25 kg). The situation is illustrated in Figure 1. In our case, the minimum dead load is equal to the minimum load (E_min_ = D_min_). The actual load-cell measuring range will be from 1.25 kg to 2.85 kg. A total of 1.6 kg of live load per load cell is taken because of a slight non-symmetry in the appliance’s construction.

In addition, the size of the load cell should not exceed 25 mm × 25 mm × 25 mm; the non-linearity and hysteresis error should not exceed 0.5%; and the sensitivity should be at least 5 times greater than in the case of a load cell with a strain-gauge element, *i.e.*, 2 mV/V on FS [7].

### 2.2. FEM simulations of the hydraulic load cell

A hydraulic load cell complying with the above requirements was conceived and modelled with the FEM prior to assembly and testing the samples in order to select the most suitable components and their dimensions. The requirements of a simple construction and standard components that can be easily assembled led to the decision to use hydroformed metallic bellows, which are usually made of a variety of materials, like stainless steel, phosphor bronze, brass, and Monel (nickel-copper alloy). In our case phosphor bronze (CuSn8) was selected. This material has excellent resistance to corrosion and is relatively free from creep, drift and hysteresis, and can be easy soldered. The fluid that fills the internal space of the hydraulic load cell is in contact with all the metallic parts and the membrane of the silicon pressure sensor. Therefore, the fluid must have good dielectric properties; it must be chemically unreactive and non-abrasive; and it must have stable physical properties over a broad temperature range. A silicon fluid was chosen because of the direct contact with the unprotected silicon pressure-sensor die, in spite of the fluid’s high volumetric coefficient of thermal expansion (CTE). Table 1 contains the material data used in the FEM.

**Figure 2.**
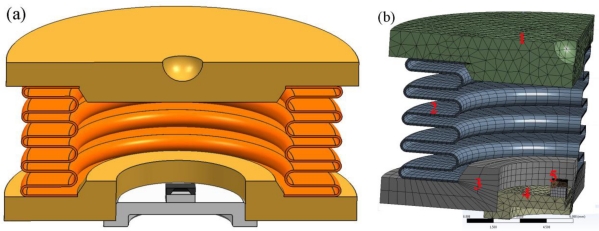
**(a)** A 3D cross-section view of the modelled hydraulic load cell**. (b)** FEM model.

A 3D model of the hydraulic load cell was created in the desired size and shape. A 3D cross-section view of the modelled hydraulic load sensor is shown in Figure 2a. Since the hydraulic load sensor is symmetrical a simulation can be made based on ¼ of the complete sensor. The situation is shown in Figure 2b with the following parts indicated: 1––upper plate, 2––hydroformed metal bellows, 3––base plate of hydraulic load, 4––T039 housing, and 5––silicon pressure sensor.

The most critical issue was to generate the mesh of finite elements, especially for the thin walls of the hydroformed metallic bellows. Some details that have no significant effect on the final result were simplified (*i.e.*, the leads of the TO-39) in order to reduce the simulation time. All the parts except the fluid were imported as a 3D model and meshed in the ANSYS Workbench environment. To create a high-quality mesh, an advanced method for meshing with imposed restrictions was used [8]. The hydroformed metallic bellows was meshed with four elements across the thickness of the wall. A ¼ of such a model consists of 19,346 elements that have 66,331 nodes.

A complementary macro describing the behaviour of the sensor filled with fluid was implemented in the ANSYS Parametric Design Language (APDL). The corresponding mesh is created with the following input parameters: thermal expansion of the fluid, the bulk modulus and the known dimensions. The resulting model enables us to calculate the mechanical deformation of the hydraulic load cell when a load is applied, the pressure change in the fluid and the output voltage of the developed sensor. Simulations of the mechanical loads at different temperatures can be performed.

**Figure 3.**
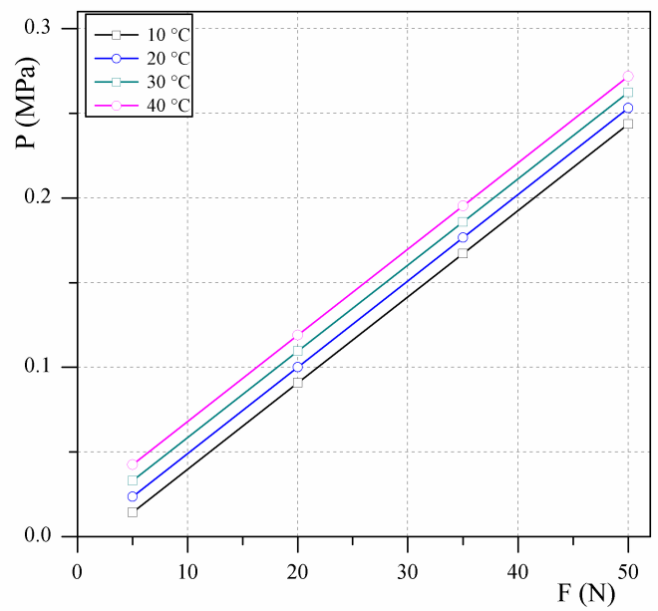
Pressure inside the hydraulic load cell at various loads and temperatures for a metallic bellows of wall thickness 0.127 mm.

We simulated three different hydroformed metal bellows made from beryllium copper CuSn8 with an outside diameter of 19.2 mm, an inside diameter 12.1 mm and wall thicknesses of 0.1 mm, 0.127 mm and 0.15 mm. Each model was loaded with a simulated 5 N, 20 N, 35 N and 50 N and at 10 °C, 20 °C, 30 °C and 40 °C. The FEM simulation results for the 0.127-mm-thick metal bellows are shown in Figure 3. The pressure inside the hydraulic load cell and its dependence on various loads at various temperatures is presented. The influence of the temperature on the internal offset pressure is significant. However, since the characteristic curves are parallel the effect of the temperature can be compensated for by software.

Simulations show that for the maximum measuring range (D_max_) the internal pressure does not exceed 0.18 MPa. Since the hydroformed metal bellows is designed for 0.25 MPa a short loading at the maximum capacity E_max_ is not expected to cause problems.

**Figure 4.**
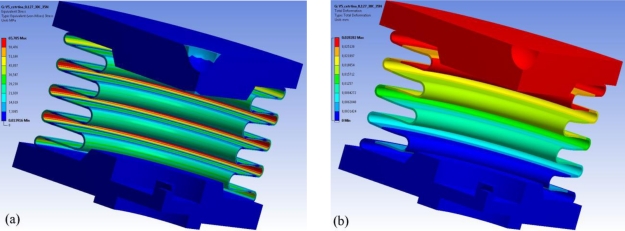
**(a)** Stress on the components of the hydraulic load cell at 30 °C and a 35 N compressive forces, with a 0.127-mm-thick wall for the hydroformed bellows, **(b)** and the corresponding deformation.

The stress, the strain and the deformation in the components of the hydraulic load cell were also simulated; the results are presented in Table 2. The simulations show that the stress in the upper plate and the base plate of the hydraulic load cell can be neglected. This means that further optimization of these two elements is possible in order to save material and reduce costs. However, this optimisation remains as a subject for future research. The stress and strain in the hydroformed metal bellows are no greater then 108 MPa and 0.98 × 10^−3^ mm/mm, respectively. The hydraulic load cell is also very stiff, which means that a deformation of no more than 0.092 mm can be expected. In Figure 4a the equivalent Von Mises stress is presented for a selected metal bellows, and the deformation is shown in Figure 4b. Deformations at 10 °C and 20 °C are negative since the reference temperature is 22 °C.

## 3. Prototypes of the Hydraulic Load Cell

### 3.1. Implementation of the prototypes

**Figure 5.**
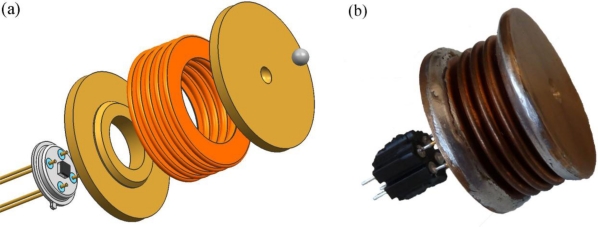
**(a)** Components of the load-cell assembly, and **(b)** implemented hydraulic load cell.

Based on the performed simulations, a series of prototypes was implemented. In the prototype phase, the metallic parts were hand soldered. The components of the hydraulic load cell are presented in Figure 5a and a sample photograph of the implemented hydraulic load cell is presented in Figure 5b.

A 0.4 MPa absolute silicon pressure-sensor die MS7904A from Intersema Sensoric was selected as the sensing device for measuring the pressure of the fluid inside the hydraulic load cell. Some of the typical characteristics of this silicon pressure-sensor die [9] are shown in Table 3.

**Figure 6.**
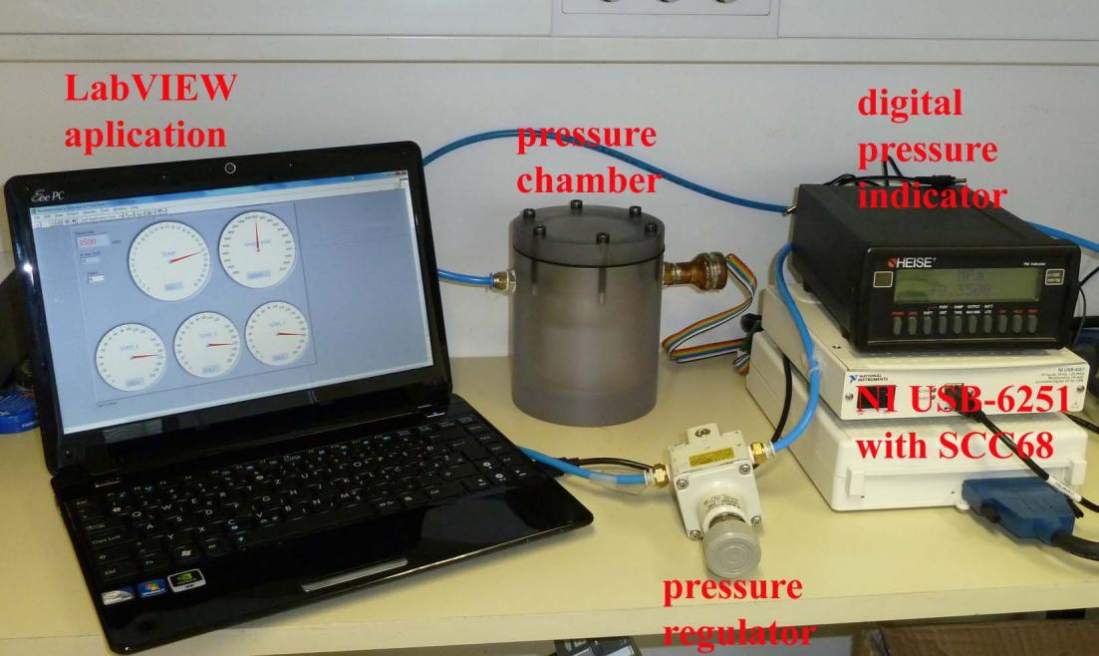
Measuring environment for the characterization of the pressure-sensor elements.

**Figure 7.**
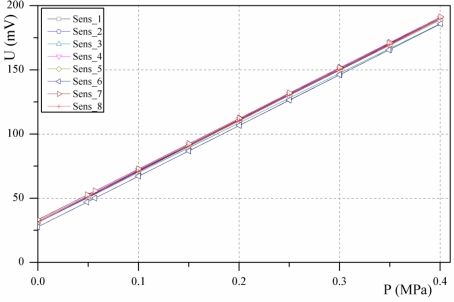
Output voltage of the pressure-sensor elements for a 5 V supply.

The silicon pressure-sensor element with dimensions of 1.58 mm × 1.72 mm × 0.91 mm was glued and bonded onto a TO-39 transistor header. To associate the measurements and the simulation results, each silicon pressure sensor die bonded on the TO-39 housing was characterized prior to the assembly of the hydraulic load cell. The pressure sensors were submerged in silicon oil and the output voltage over the entire pressure range was measured in a pressure chamber. Using the pressure regulator (SMC IR 2000-F02) and the digital pressure indicator (HEISE PM) the desired pressure was adjusted. The measuring environment is shown in Figure 6. The output voltage *versus* the applied pressure for a 5 V supply to the silicon pressure sensor is presented in Figure 7. The average value of the offset output voltages was 30.2 mV and the calculated sensitivity for the sensors was about 392.93 mV/MPa. The analysis of the measurements shows that the non-linearity and the hysteresis errors are in the declared range specified by the producer.

The hydraulic load cell was assembled as follows. The upper plate and the base plate were machined from the CuSn12 phosphor bronze rod. In the centre of the base plate a hole of 7.7 mm was drilled, into which the TO-39 transistor header with the pressure-sensor element was fitted. An additional hole of 2 mm diameter was employed in order to be able to fill the hydraulic load cell with fluid. In the centre of the upper plate there is a place for a 2.4 mm steel ball. In this way, the influence of side loading is minimized. A hydroformed metallic bellows made from beryllium copper CuSn8 was used. The metallic bellows was taken from the standard product range of the producer Hydeoflex. The bellows consist of five convolutions with an outer diameter of 19.1 mm, an inner diameter of 12.4 mm and declared wall thickness of 0.127 mm.

The interior of the hydraulic load cell was completely filled with Wacker AK-100 silicon oil. To prevent air bubbles, the samples were filled in a vacuum chamber where an absolute pressure of 0.01 Pa was established. At the end of the filling process the hole in the base plate was sealed to prevent any leakage of fluid from the hydraulic load cell. An additional pin connector for attaching a measurement system is soldered onto the pins of the TO-39 transistor header.

### 3.2. Characterization of the prototypes

The important characteristics of the hydraulic load cells in the produced prototypes, such as the non-linearity, the hysteresis error, the sensitivity and the repeatability, were measured. The above terms will now be described in more detail. Non-linearity is the deviation from a straight line for the increasing output of the sensor signal curve. For this benchmarking, a linear approximation between the first and the last measured points was taken. The hysteresis error is defined as the difference between the load-cell readings for the same applied load: one reading obtained by increasing the load from the minimum load and the other by decreasing the load from the maximum load. The sensitivity is the ratio of the change in the response (output) of a load cell to the corresponding change in the stimulus (applied load). The repeatability is the ability of the load cell to provide successive results that are in agreement when the same load is applied several times and applied in the same manner on the force sensor under constant test conditions.

**Figure 8.**
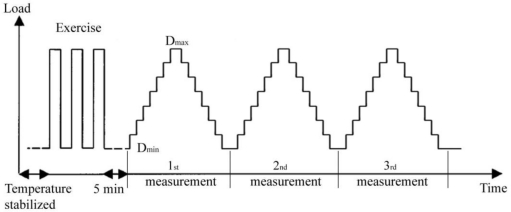
Test sequence for each temperature.

The samples were characterized at 10 °C, 20 °C, 30°C and 40 °C. The measurements were made according to the OIML recommendation [6]. A test sequence with the so-called exercise procedure before the measurement is shown in Figure 8. After temperature stabilization, the load cell was exercised by applying the maximum test load D_max_ three times, and then returning to the minimum test load D_min_ after each load application. Each sample was measured three times in a row, 5 minutes after the exercise procedure. Increasing loads were applied from the minimum test load D_min_ to the maximum test load D_max_, and then back to D_min_. The measurement of the sensor output voltage was made 10 seconds after applying the load at each of the 8 test points for increasing and decreasing test loads. The average values of three measurements were calculated and processed in subsequent calculations.

**Figure 9.**
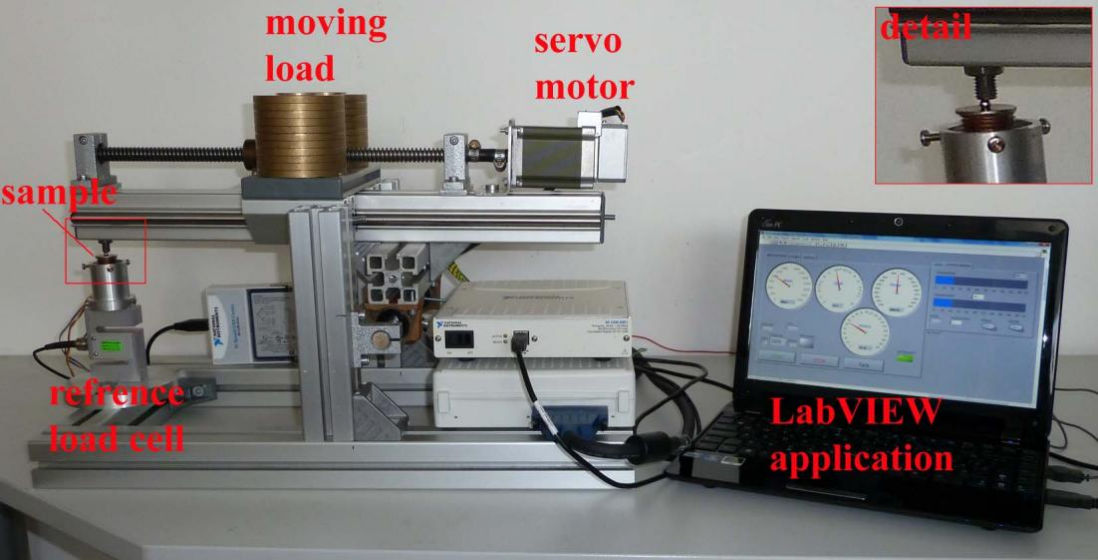
Measurement system for characterization of the hydraulic load cells. A detail of the test sample on the special holder is shown in the top-right-hand corner.

To achieve more accurate and repeatable tests, a special measurement device for characterising the hydraulic load cell (shown in Figure 9) was built. The whole procedure for the exercise and the measurements of the test samples was controlled and measured in the LabVIEW environment. The load on the linear slide is moved back and forth by a servo motor; this then creates the load on the tested sample, which is placed on a special holder. A calibrated reference load cell is used for measuring the actual load on the tested sample. National Instruments data-acquisition cards (DAQ) were used: a 24-bit NI USB-9327 for the reference load cell and a 16-bit NI USB-6251 for measuring the hydraulic load cell. A WEISS WK1-180 climate chamber was used for the measurements in the controlled temperature environment. During the measurements the measurement system, except for the DAQ cards and the portable computer, was placed in a climatically controlled chamber. During the measurement of the non-linearity and hysteresis, the compressor and the ventilator of this chamber were switched off to reduce the influence of vibrations on the measurement.

The measurements were performed in such a way that the target environment was simulated. Four hydraulic load cells were placed at the bottom of the appliance and loaded with the weight of the appliance. The hydraulic load cell was loaded with the minimum load (D_min_) of 1.25 kg. The load cell’s measuring range is 1.6 kg, which means that the maximum load (D_max_) is 2.85 kg. In the above measurement configuration, the measurements parameters can be easily adjusted using the software.

## 4. Results and Discussion

**Figure 10.**
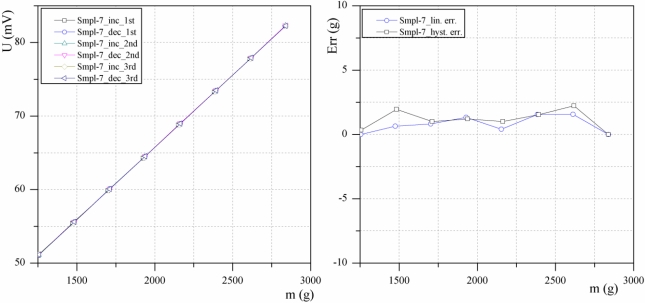
**(a)** Output voltage *versus* mechanical load of the sensor––three times repeated measurement and **(b)** non-linearity error and hysteresis error.

Figure 10a presents typical results for samples measured three times. Sample 7 was measured three times in a row in a temperature chamber at 20 °C for increasing and decreasing load. The resulting measurements are close to each other so that the six plots nearly coincide and cannot be distinguished in the figure. In Figure 10b the average deviations of the three measurements from the ideally linear response of the sensor are shown. As can be seen from the diagram, the absolute error does not exceed 3 grams (0.3%) and the hysteresis error is about 1 gram (0.2%). Other samples exhibited similar characteristics. The hysteresis error is small, and this can be reduced by using similar methods to those used in the strain-gauge load cells [10].

**Figure 11.**
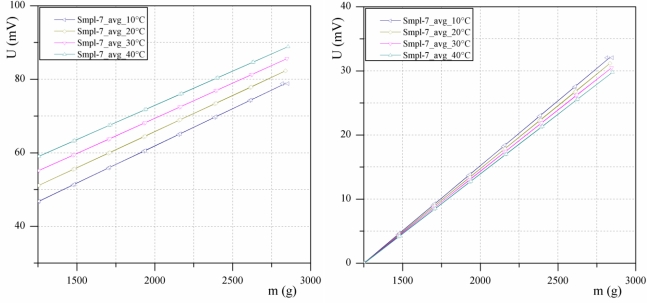
**(a)** Output voltage *versus* mechanical load of the sensor at different temperatures and **(b)** the same results using the tare function.

The samples were characterized at different temperatures, *i.e.*, 10 °C, 20 °C, 30 and 40 °C. The average values of the three measurements for each temperature are shown in Figure 11a. As expected from the FEM simulations, the temperature has an influence on the offset of the output curve. However, different offsets can be easily compensated for by using the tare function. Nevertheless, a slight problem remains. Figure 11b shows the output curves compensated for by the tare function. As can be seen, the curves deviate at higher loads. This means that an error of 2.2 mV at D_max_ can be expected, which represents an approximately 7.5% error over the entire temperature range of 30 °C. Such a strong temperature dependence must be compensated for in order to reduce the final error to the desired level. Preliminary estimations show that the temperature error can be reduced by software compensation. In this respect, pressure sensor dye with integrated temperature element can be used for measuring pressure and temperature.

The comparison of the designed prototype with other load cell candidates for the target application is given in Table 4. Typical representatives of different types of load cells were considered. Conventional strain-gauge based load-cells with double bending spring element exhibit small non-linearity and hysteresis error. However, their sensitivity is low and even the cost may prove to be prohibitive for the target application. In addition, oversized dimensions impose restrictions on miniaturization of the target kitchen appliance. Thick-film based load cells have better sensitivity and acceptable price but still impose problems due to their size. Silicon based load cells have high sensitivity and desired dimensions but their maximum capacity is too low. Furthermore, their price is too high (*i.e.*, the four load cells would cost more than the appliance is worth).

In general, none of the compared products sufficiently fulfils the requirements which fostered the development of the reported hydraulic load cell. The resulting load sell is composed of standard machine parts and its dimensions allow it to be integrated in the feet of the appliance. Its small dimension, low cost and relatively high sensitivity offer an attractive alternative to the devices currently used in similar applications. As regards robustness, its safe load limit is comparable to the other load cells in Table 4.

## 5. Conclusions

In this paper a hydraulic load cell of simple construction for measuring small loads is presented. Particular care was taken to design the load cell with standard components. In addition, design-for-manufacturing issues were also of prime concern. The high response of the output voltage of the developed device ensures good noise immunity and gives it an advantage over conventional strain-gauge load cells. Also additional improvements are possible [11]. The main characteristics were measured in accordance with the OIML recommendation, and the obtained results fulfil the requirements imposed by the target application. As shown in Table 4, the developed load cell exhibits small dimensions, low hysteresis and nonlinearity error, high sensitivity and acceptable production cost, while other candidate products proved inadequate in some respects (*i.e.*, desired technical parameters and/or price).

In the future we plan to further decrease the influence of temperature on the measurement characteristics by using a fluid with a lower TCE. In this respect, various mixtures of fluids (*i.e.*, [12]) are currently being investigated. Furthermore, the remaining error will be reduced by employing software compensation. However, in order to do this, the temperature within the sensor must be known. This will require the use of a silicon pressure-sensor die with an integrated temperature-sensing element [13].

## Figures and Tables

**Table 1.** Material data used in the simulations of the hydraulic load cell.

**Material**	***E* (GPa)**	***γ***	***α* (×10^−6^ /K)**	***K* (GPa)**	***β* (×10^−4^ /K)**
CuSn12	105	0.3	18	/	/
CuSn8	110	0.34	18.2	/	/
Steel	200	0.3	10.4	/	/
Silicon	165	0.22	3.5	/	/
Silicone fluid	/	/	/	1	9.4

*E* ––Young’s modulus; *γ* ––Poisson’s ratio; *α* ––linear coefficients of thermal expansion; *K* ––bulk modulus; *β* ––volumetric coefficients of thermal expansion.

**Table 2.** Simulated data for the von Mises stress, von Mises strain and the total deformation.

**F (N)**	**T (°C)**	**Stress (MPa)**	**Strain (mm/mm)**	**Deformation (mm)**
5	10	30.4	2.76E−04	−6.41E−02
20	10	53.0	4.82E−04	−7.01E−02
35	10	75.6	6.88E−04	−7.61E−02
50	10	98.7	8.97E−04	−7.32E−02
5	20	11.3	1.02E−04	−1.22E−02
20	20	34.3	2.77E−04	−1.89E−02
35	20	57.4	5.21E−04	−2.56E−02
50	20	80.5	7.32E−04	−3.25E−02
5	30	25.5	2.31E−04	3.95E−02
20	30	45.1	4.10E−04	3.39E−02
35	30	65.8	5.89E−04	2.83E−02
50	30	84.5	7.68E−04	2.35E−02
5	40	49.2	4.48E−04	9.15E−02
20	40	68.9	6.26E−04	8.58E−02
35	40	88.7	8.06E−04	8.06E−02
50	40	108.0	9.83E−04	7.46E−02

**Table 3.** Some typical characteristics of the MS7904A silicon pressure sensor die.

**Parameter**	**Min**	**Typ**	**Max**
Operating pressure (MPa)	0		0.4
Operating temperature range (°C)	−40		125
Bridge resistance (kΩ)	3.0	3.4	3.8
Full-scale span (FS) (mV)	120	150	180
Zero pressure offset (mV)	−40	0	40
Linearity (% FS)		±0.05	±0.15
Hysteresis (% FS)		±0.05	±0.15
Temperature coefficient of offset (μV/°C)	−80		+80

**Table 4.** comparison with other load cell candidates for the target application.

	**Strain-gauge based load cell**	**Thick-film strain-gauge load cell**	**Silicon based load cell**	**Hydraulic load cell**
Device	K-DFTA 5KGVOR3-1	Laboratory sample	FSS 1550	Prototype
Max. capacity (E_max_) (kg)	5	5	1,5	5
Size (L×W×H) (mm)	60 × 10 × 6	80 × 13 × 12	10 × 6 × 4	Φ21 × 10
Input/output resistance (Ω)	350	1300	5,000	3500
Non-linearity error (% of FS)	±0.05	±0.2	±1.5	±0.3
Hysteresis error (% of FS)	±0.05	±0.2	/	±0.2
FS Sensitivity (mV/V)	1	1.4	36	10
Offset (mV/V)	±1	±4	±15	±60
Price	mid	low	high	low
